# Decreased Bone Mineral Density Is an Independent Predictor for the Development of Atherosclerosis: A Systematic Review and Meta-Analysis

**DOI:** 10.1371/journal.pone.0154740

**Published:** 2016-05-05

**Authors:** Chenyi Ye, Mingyuan Xu, Shengdong Wang, Shuai Jiang, Xi Chen, Xiaoyu Zhou, Rongxin He

**Affiliations:** 1 Department of Orthopedics, The Second Affiliated Hospital, School of Medicine, Zhejiang University, Hangzhou, People’s Republic of China; 2 Department of Plastic Surgery, The First Affiliated Hospital, School of Medicine, Zhejiang University, Hangzhou, People’s Republic of China; Innsbruck Medical University, AUSTRIA

## Abstract

**Background:**

There is conflicting evidence regarding the association between decreased bone mineral density (BMD) and atherosclerosis. To this end, we performed a systematic review and meta-analysis to clarify the association.

**Methods:**

To identify relevant studies, PubMed, Embase, and the Cochrane Library were systematically searched up to November 2015. All observational and comparative studies directly investigating the relationship between decreased BMD and clinical consequences of atherosclerotic vascular abnormalities, including carotid artery calcification (CAC), cardiovascular disease (CAD), and coronary artery disease (CAD) were obtained, without limitation of language or publication year.

**Results:**

A total of 25 studies involving 10,299 patients were included. The incidence of atherosclerotic vascular abnormalities was significantly increased in low BMD patients, compared to patients with normal BMD (OR, 1.81, 95% CI [1.01, 2.19], p<0.00001)). Similar results were also observed for postmenopausal women (OR, 2.23, 95% CI [1.72, 2.89], p<0.00001). Subgroup analyses of osteopenia, osteoporosis, and normal BMD also revealed that the combined ORs for the incidence of atherosclerotic vascular abnormalities increased as BMD decreased. Of note, after adjusting for age, sex, body mass index (BMI) and other vascular risk factors, decreased BMD remained significantly associated with the incidence of atherosclerotic vascular abnormalities (OR, 2.96, 95% CI [2.25, 3.88], p < 0.00001).

**Conclusions:**

Based on the results of this study, decreased BMD is an independent predictor for the development of atherosclerosis in elderly individuals. Moreover, the risk of atherosclerotic vascular abnormalities increased as BMD decreased. Future studies focusing on individuals with different severities of atherosclerosis and comorbidities are of interest.

## Introduction

Osteoporosis (OP) and atherosclerosis are the two most common diseases in elderly individuals and are associated with significant morbidity, mortality, and disability. OP affects 44 million Americans (55%) over the age of 50 years [1, 2]. According to data from the World Health Organization (WHO), elderly individuals will comprise 20% of the population by 2050 [3–5], increasing the prevalence of OP and atherosclerosis.

Although mounting epidemiological evidence suggests an association between OP and atherosclerosis, these diseases are traditionally considered unrelated, with their co-existence being caused by independent age-related processes. Recently, a growing number of studies have linked atherosclerosis to OP in elderly individuals [3, 4, 6–13]. The association, however, is not fully understood and controversy still exists regarding whether it is gender-related. Moreover, the connection between bone mineral density (BMD) and atherosclerosis is also controversial. Several studies suggested that no relationship exists between decreased BMD and atherosclerosis [9, 14–18]. In another study, osteoporosis and atherosclerosis were independent processes in postmenopausal women, after adjusting for age, gender, BMI, hypertension, and other vascular risk factors [4]. Beer *et al*. also revealed that low BMD was not associated with coronary atherosclerosis in men [5].

In the absence of any studies that are sufficiently powered to examine the relationship between low BMD and atherosclerosis, a meta-analysis of previously conducted investigations is useful. Thus, we conducted a systematic review and meta-analysis of previous studies for the following purposes: (1) to investigate and compare the incidence of atherosclerotic vascular abnormalities in individuals with normal BMD, osteopenia, and osteoporosis; (2) to explore the relationships between low BMD and atherosclerotic vascular abnormalities among the complete population and in postmenopausal women; and (3) examine the relationships after adjusting for age, sex, body mass index (BMI) and other vascular risk factors.

## Methods

This study was performed in accordance with the Preferred Reporting Items for Systematic Reviews and Meta-Analyses (PRISMA) guidelines [19], and the Cochrane Handbook for Systematic Reviews of Interventions (ver. 5.0.2).

### Literature search

An experienced medical librarian designed and performed the literature search. Three independent investigators (CY, MX, SW) searched electronic databases (PubMed, Embase, and the Cochrane Library) with no language restrictions through November 2015. The following keywords or corresponding Medical Subject Headings (MeSH) were used: “bone mineral density” or “BMD” or “osteoporosis” or “OP” or “osteopenia” and “atherosclerosis” or “atheroscleroses” or “calcium plaque” or “carotid artery calcification” or “CAC” or “cardiovascular disease” or “CVD” or “coronary artery disease” or “CAD” or “atherosclerotic vascular disease” or “AVD” or “coronary micro-vascular endothelial dysfunction” or “CMED”. Details of the search strategy are provided in S1 Table and S1 and S2 Texts. Reference lists were also searched manually for additional studies. In addition, we searched the National Institutes of Health, the Clinical Trial Registry, the Trials Central and the Center Watch, and the Current Controlled Trials for grey literature.

### Inclusion criteria

Two reviewers (CY and MX) independently screened manuscript titles and abstracts, and implemented the following inclusion criteria: (1) observational studies that examined the association between low BMD and clinical consequences of atherosclerotic vascular abnormalities, including carotid artery calcification (CAC), cardiovascular disease (CAD), and coronary artery disease (CAD); (2) studies that enrolled individuals aged 18 years and older; and (3) studies that provided data pertaining to BMD in participants with and without atherosclerotic vascular abnormalities.

The following exclusion criteria were used in this study: (1) articles that did not satisfy the inclusion criteria; (2) animal studies, reviews, letters, abstracts, case reports, conference proceedings, and systematic reviews; and (3) studies that did not provide sufficient data on BMD values, including the means, medians, standard deviations (SDs), and/or standard errors for subjects with and without atherosclerotic vascular abnormalities. Any disagreements were evaluated using a kappa test and consensus was achieved by discussion with the corresponding author (RH).

### Data extraction and quality assessment

Three independent reviewers (SJ, XC, XZ) obtained relevant data and assessed the accuracy. The following information was extracted from each study: first author’s family name, year of publication, country, study design, patient demographics (age, gender, sample size), BMD values, assessment methods, and definition of atherosclerotic vascular abnormalities. Contact with corresponding authors was also attempted to verify the accuracy of the data, as well as to obtain further data for the analysis. The quality of the methodological data included in the studies was assessed using the Newcastle-Ottawa Scale (NOS), as recommended by the Cochrane non-randomized studies methods working group [20]. All articles were scored according the three major categories of NOS (selection, comparability andassessment of outcome). The maximum scores for selection, comparability and exposure were 4, 2 and 3, respectively. Studies were considered of high quality if at least five of the nine criteria were met.

### Statistical analysis

Revman software (ver. 5.3; The Nordic Cochrane Centre, Copenhagen, Denmark) was used to pool the data. A p-value ≤ 0.05 was considered significant. Summary odds ratio (OR) and its 95% CI were calculated to assess the association between low BMD and the occurrence of atherosclerotic vascular abnormalities across studies. For continuous data, the SD was calculated using the method described by Walter *et al*. [21]. Fixed- and random-effects models were used to calculate the combined ORs. Q and *I*^*2*^ statistics were used to assess the heterogeneity of the ORs across multiple studies [22]. A p-value < 0.10 was considered significant. If no statistical heterogeneity was detected between studies (P > 0.10; *I*^*2*^ < 50%), a fixed-effect test (Mantel-Haenszel test) was used to pool the data. Otherwise, the random-effect (Der Simonian-Laird method) model was applied. A sensitivity analysis was also performed to explore possible explanations for heterogeneity.

Subgroup analyses were conducted according to the severity of low BMD and gender, to evaluate the potential effect on outcomes of modifying these variables. The symmetry of funnel plot was also employed to evaluate publication bias. Furthermore, Egger’s test and Begger’s test were performed to assess the publication bias.

## Results

### Literature search

**Fig 1:**
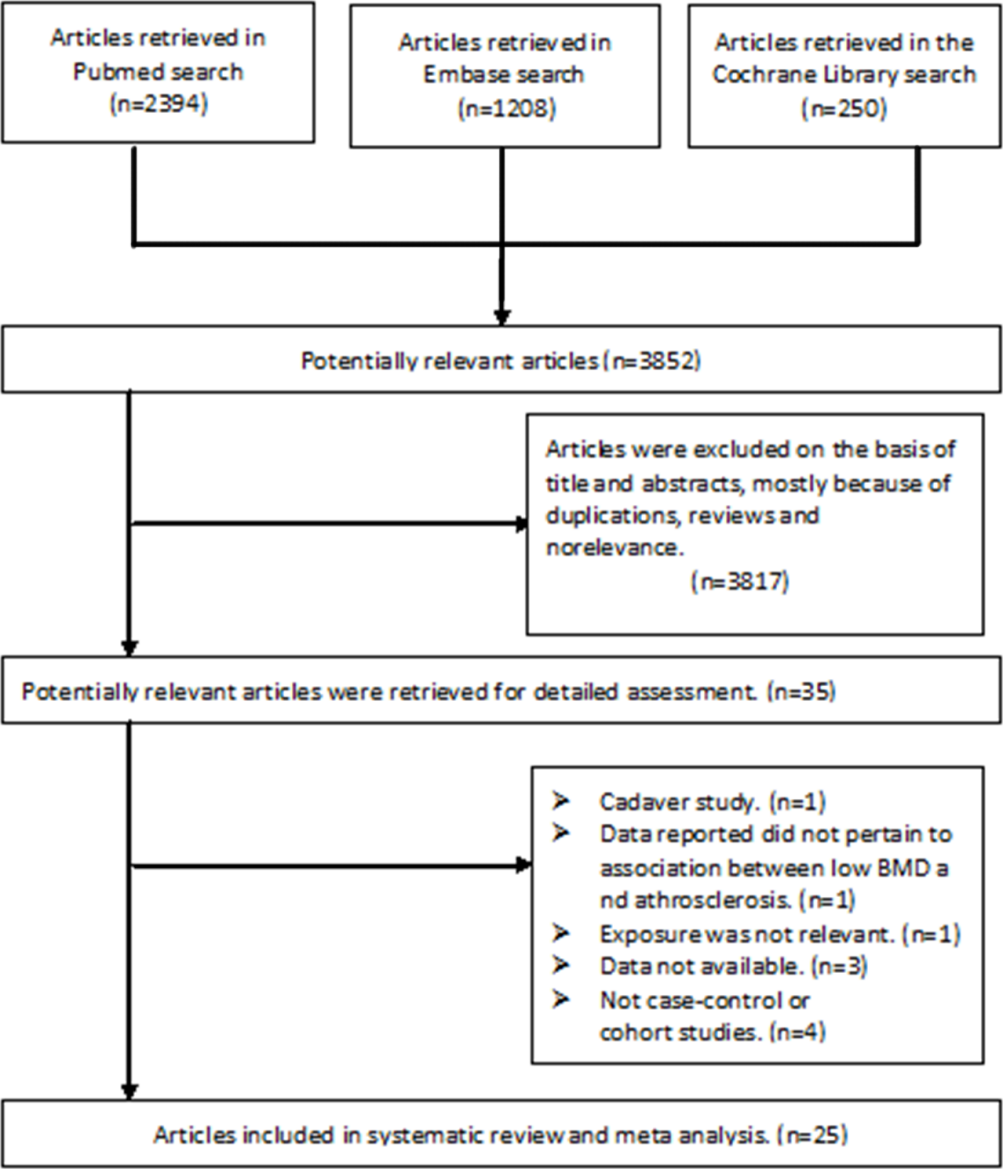
A PRISMA flowchart illustrated the selection of studies included in our systematic review.

In total, the search identified 3,852 candidate publications; however, 3,817 were excluded due to duplications, nonrelevance, or because they were not observational or comparative studies. After assessing the 35 potentially-relevant articles, 25 articles (23 case-control studies and 2 cohort studies) involving 10,299 patients met the inclusion criteria [5, 15–18, 23–42]. The primary reasons for exclusion were as follows: one paper was a cadaver study [43]; one study failed to relate the data to low BMD and atherosclerotic vascular abnormalities [44]; one study was based on male patients with with type 2 diabetes mellitus, and the exposure was not relevant [45]; three articles were excluded because the study population size was unavailable and the association between low BMD and atherosclerosis was not presented [9, 46, 47]; and four studies were excluded because they were not case-control or cohort studies [12, 48–50]. The details of study selection are presented in Fig 1. The weighted kappa for the agreement on eligibility between the reviewers was 0.87 (95% CI [0.82–0.91]).

### Study characteristics

The characteristics and quality assessment results of the 25 studies are summarized in Table 1. Of those, two studies were performed in Italy, six in the US, three in China, three in Japan, two in Turkey, two in Korea, one in Morocco, one in Romania, one in Poland, one in Austria, one in Iran, one in Egypt, and one in Finland. Participants ranged in age from 46 to 89 years, and five of the studies included both males and females [5, 18, 24, 26, 37]. Nineteen studies included only post-menopausal individuals and one only consisted of male participants [15–17, 23, 25, 28–30, 32–35, 37, 39–42].

**Table 1:** Characteristics of subjects in eligible studies.

			Size					Gender					
Study	Country	Study design	Total	Normal BMD	Low BMD	Osteopenia	Osteoporosis	M	F	Age	Measurement of AS	Results	NOS Score
Montalcini, 2004 [15]	Italy	case-control	157	100	57	NA	NA	0	157^a^	56±8	CAC	-	5
Tankó, 2005 [23]	America	case-control	2576	NA	2442	1153	1289	0	2576^a^	67±7	CVD	+	7
Marcovitz, 2005[24]	America	case-control	209	52	157	90	67	26	183	67±11	CAD	+	6
Gupta, 2006 [25]	America	case-control	101	50	51	NA	NA	0	101^a^	77±12	AVD	+	6
Varma, 2007 [26]	America	case-control	198	66	132	79	53	52	146^a^	66±6	CAD	+	6
Tekin, 2008 [16]	Turkey	case-control	227	57	159	NA	NA	0	227^a^	60±9	CAD	-	7
Sumino, 2008 [27]	Japan	case-control	175	43	132	73	59	0	175^a^	59±9	CAC, cIMT	+	7
Tamaki, 2008 [28]	Japan	cohort	609	71	88	66	22	0	609^a^	56±10	CAC	+	5
Reddy, 2008 [29]	America	case-control	228	68	160	95	65	0	228^a^	64±10	CVD, BAC	+	7
Hmamouchi, 2009 [30]	Morocco	case-control	72	NA	NA	NA	40	0	72^a^	59 ± 8	CAC, cIMT	±	7
Mikumo, 2009 [31]	Japan	case-control	143	17	21	12	9	0	143^a^	58 ± 8	AVD, PWV	+	7
Seo, 2009 [32]	Korea	case-control	152	35	117	86	31	0	152^a^	56 ± 6	CAD, PWV	+	5
Pennisia, 2010 [33]	Italy	case-control	100	20	72	32	40	0	100^a^	75±10	CAC, Femoral AS	±	6
Fodor, 2011 [34]	Romania	case-control	100	36	64	32	32	0	101^a^	65±10	CAC, cIMT	+	7
Bajew, 2011 [17]	Poland	case-control	61	23	38	22	16	0	61^a^	68±9	CAD	-	5
Beer, 2011 [5]	Austria	case-control	623	351	272	207	65	623	0	64±11	CAD	-	7
Hajsadeghi, 2011 [18]	Iran	case-control	119	25	94	39	55	58	61	59±8	CAD	-	5
Shokry, 2012 [35]	Egypt	case-control	100	30	NA	NA	30	0	100^a^	63±4	CAD, PAD	+	7
Yesil, 2012 [36]	Turkey	case-control	2235	174	2061	766	1335	827	1408^a^	73±6	CVD	+	7
Teng, 2013 [37]	China	case-control	481	163	318	155	163	0	481^a^	72±6	AVD, WBV	+	6
Liang, 2014 [38]	China	case-control	385	206	179	130	49	163	222	59±12	CVD, PWV, cIMT	±	5
Prasad, 2014 [39]	America	cohort	194	NA	NA	NA	39	0	194^a^	61±7	CAD, CMED	+	7
Värri, 2014 [40]	Finland	case-control	290	122	168	148	20	0	290^a^	74±3	CAC, cIMT	±	6
Seo, 2015 [41]	Korea	case-control	252	75	177	NA	NA	0	252^a^	55±6	CAD	+	7
Yu, 2015 [42]	China	case-control	512	NA	NA	NA	204	0	512^a^	75±5	AVD, PWV	+	6

CAC: carotid artery calcification. CVD: cardiovascular disease. CAD: coronary artery disease. AVD: atherosclerotic vascular disease. IMT: intima-media thickness. BAC: breast arterial calcification. PWV: pulse wave velocity. AS: atherosclerosis. PAD: peripheral arterial disease. cIMT: Carotid IMT. WBV: whole blood viscosity. CMED: coronary microvascular endothelial dysfunction. Normal BMD: T-score>-1; Osteopenia: -1<T-score<-2.5; Osteoporosis: T-score<-2.5. + positive correlation between BMD and outcomes; − negative correlation between BMD and outcomes; ± both positive and negative correlation between BMD and outcomes were reported.

^a^ postmenopausal.

The definition of arthrosclerotic vascular abnormalities was determined by the included studies, which referred to: carotid artery calcification (CAC); cardiovascular disease (CVD); and coronary artery disease (CAD). The pulse wave velocity (PWV), breast arterial calcification (BAC), intima-media thickness (IMT) and whole blood viscosity (WBV) were also recorded whenever available. The definition of normal BMD, osteopenia, and osteoporosis was made according to the criteria of the WHO. Osteoporosis was defined as BMD minus 2.5 standard deviations (SD) (or lower than the young adult mean), while osteopenia was defined as BMD between 1 and 2.5 SD below the young adult mean [51]. Low BMD was defined as a combination of osteoporosis and osteopenia. Accordingly, 1,784 individuals were included in the normal BMD group, while 6,959 individuals were included in the low BMD group. There were 3,185 individuals in the osteopenia group, and 3,683 individuals were included in the osteoporosis group.

Seven studies comprised of 5,850 participants were adjusted for age, gender, BMI, hypertension, and other vascular risk factors [23, 24, 29, 36, 39–41]. The adjusted ORs with 95% CIs are listed in Table 2. The overall quality of the studies averaged a score of 6 points (range: 5–7 points) on a scale of 0 to 9.

**Table 2:** Characteristics of eligible studies after adjusting for covariates.

Study	Total Size	Gender	Comparison	Adjusted RR (95%CI)	Adjustment for covariates	Conclusion
Tankó, 2005 [23]	2442	F	Osteoporosis vs Osteopenia	3.90 (2.00–7.70)	Age, Sex, diabetes, hypertension, hyperlipidemia, smoking, and prior CHD events.	Women with osteoporosis had increased risk for CV events.
Marcovitz, 2005 [24]	209	M/F	Osteoporosis vs Osteopenia	5.58 (2.59–12.0)	Age, Sex, BMI, and hypertension, hyperlipidemia, smoking, and prior CHD events.	Low BMD predict significant CAD in women.
Reddy, 2008 [29]	228	F	Osteopenia vs Normal BMD	2.70 (1.10–6.80)	Age, Sex, BMI, and other vascular risk factors (Menopause, Diabetes mellitus,Hypertension).	Osteoporosis was strongly associated with the presence of BAC.
			Osteoporosis vs Normal BMD	4.40 (1.60–12.0)	Age, Sex, BMI, and other vascular risk factors (Menopause, Diabetes mellitus,Hypertension).	Osteoporosis was strongly associated with the presence of BAC.
Yesil, 2012 [36]	2235	M/F	Low BMD vs Normal BMD	1.64 (1.07–2.53)	Age, Sex, BMI, and Diabetes mellitus, Hypertension, Smoking, TC.	Significant negative correlation between CAD and OP/osteopenia.
Prasad, 2014 [39]	194	F	Osteoporosis vs Non- osteoporosis	2.40 (1.10–5.60)	Age, hyperlipidemia, hypertension, BMI, estrogen use, and steroid use.	Women with CMED were twice as likely to develop osteoporosis.
Värri, 2014 [40]	290	F	Osteopenia vs Normal BMD	1.70 (1.00–2.90)	Age, BMI, current smoking, HT use,and systolic blood pressure.	Maximum cIMT but not mean cIMT significantly associated with low BMD.
			Osteoporosis vs Normal BMD	4.20 (1.10–15.9)	Age, BMI, current smoking, HT use,and systolic blood pressure.	Maximum cIMT but not mean cIMT significantly associated with low BMD.
Seo, 2015 [41]	252	F	Low BMD vs Normal BMD	3.35 (1.07–10.6)	Age, alcohol intake, exercise, and vascular risk factors.	Decreased BMD is associated with coronary atherosclerosis in healthy postmenopausal women.

CV events: cardiovescular events. CMED: coronary microvascular endothelial dysfunction. Normal BMD: T-score>-1; Osteopenia: -1<T-score<-2.5; Osteoporosis: T-score<-2.5. CHD: coronary heart disease. BAC: breast arterial calcification. TC: total cholesterol. HT: hormone therapy.

### Results of the population study

The pooled results are shown in Table 3.

**Table 3:** Summary of outcomes comparing BMD and the incidents of atherosclerosis.

Outcomes	Gender	Studies	Participants	Odds Ratio, 95% CI	I^2^ for Heterogeneity	p value
1. In male and female						
Osteopenia vs Normal BMD	M/F	9	1912	1.84 [1.45, 2.32]	0%	p<0.00001
Osteoporosis vs Normal BMD	M/F	9	2491	2.05 [1.55, 2.71]	41%	p<0.00001
Low BMD vs Normal BMD	M/F	14	4933	1.81 [1.01, 2.19]	49%	p<0.00001
Osteoporosis vs Non-osteoporosis	M/F	11	2238	2.45 [1.90, 3.17]	40%	p<0.00001
2. In postmenopausal women						
Osteopenia vs Normal BMD	F	5	651	2.07 [1.43, 3.00]	41%	p = 0.00001
Osteoporosis vs Normal BMD	F	6	367	4.29 [2.37, 7.77]	0%	p<0.00001
Low BMD vs Normal BMD	F	7	1305	1.23 [1.72, 2.89]	39%	p<0.00001
Osteoporosis vs Non-osteoporosis	F	7	1009	2.05 [1.13, 3.72]	61%	p = 0.02
3. Ajusted for Age, Sex, BMI and other vascular risk factors						
Low BMD vs Normal BMD	M/F	7	5641	2.96 [2.25, 3.88]	29%	p<0.00001

#### Primary results

**Fig 2:**
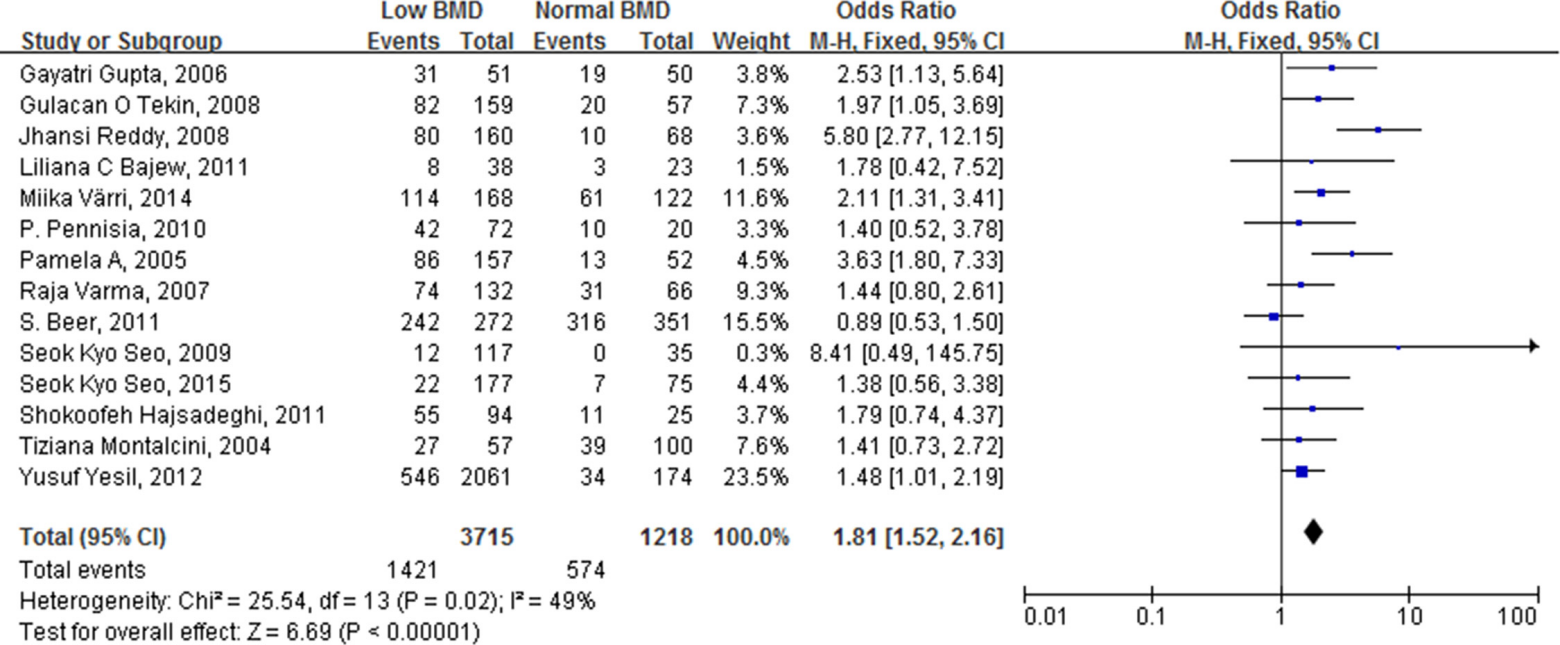
Forest plot shows that the incidence of atherosclerotic vascular abnormalities is significantly higher in individuals (including male and female) with low BMD than those with normal BMD.

The ORs for each study and the combined OR for patients with low and normal BMD are shown in Fig 2. The combined OR for the incidence of atherosclerotic vascular abnormalities in patients with low BMD, compared to patients with normal BMD, was 1.81 (95% CI [1.01, 2.19], *p* < 0.00001).

#### Subgroup and sensitivity analysis

**Fig 3:**
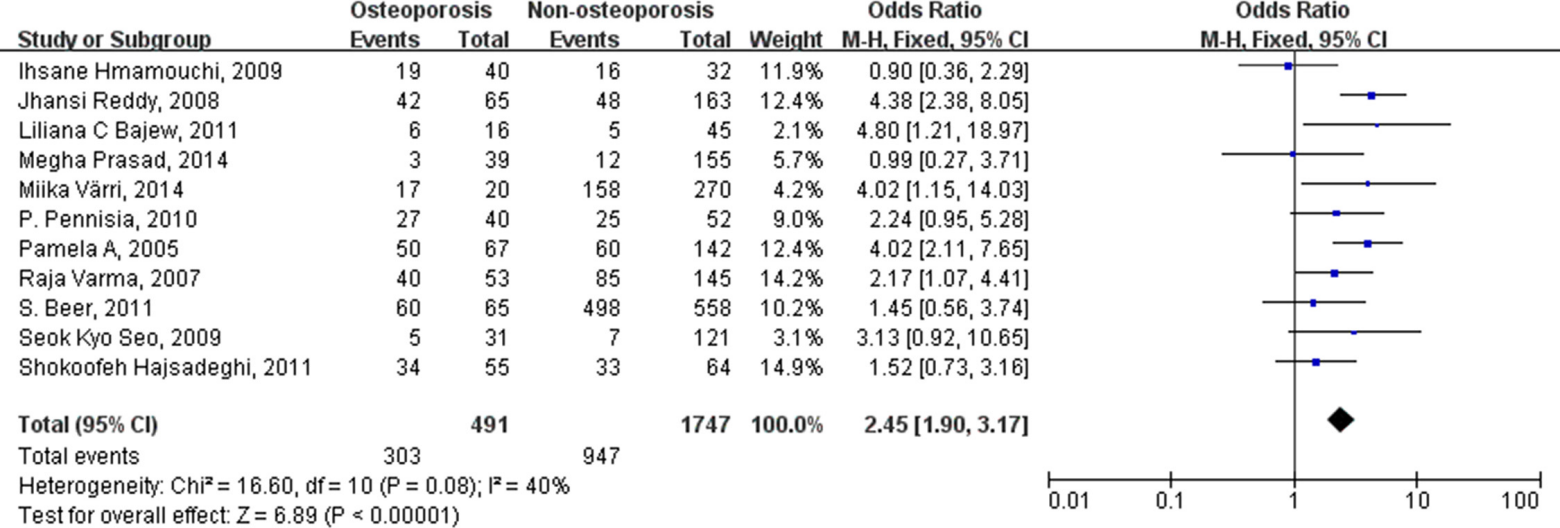
Forest plot shows that the incidence of atherosclerotic vascular abnormalities is significantly higher in individuals (including male and female) with osteoporosis than those without osteoporosis.

The results of the subgroup analyses that were based on the severity of the decreased BMD are shown in Table 3. The combined OR for the incidence of atherosclerotic vascular abnormalities in patients with osteopenia, compared to patients with normal BMD, was 1.84 (95% CI [1.45, 2.32], p < 0.00001). The combined OR for the incidence of atherosclerotic vascular abnormalities in patients with osteoporosis, compared to patients with normal BMD, was 2.05 (95% CI [1.55, 2.71], p < 0.00001). Finally, when comparing patients with and without osteoporosis, the combined OR for the incidence of atherosclerotic vascular abnormalities was 2.45 (95% CI [1.90, 3.17], p < 0.00001) (Fig 3).

No evidence of significant heterogeneity was observed within any subgroup, while a significant association between incidence of atherosclerotic vascular abnormalities and decreased BMD was observed in all subgroups.

To test the robustness of the data, sensitivity analyses were performed by omitting one study at a time. These analyses yielded similar results to those obtained when all studies were analyzed simultaneously. The pooled ORs were all statistically significant.

### Results of postmenopausal women

**Fig 4:**
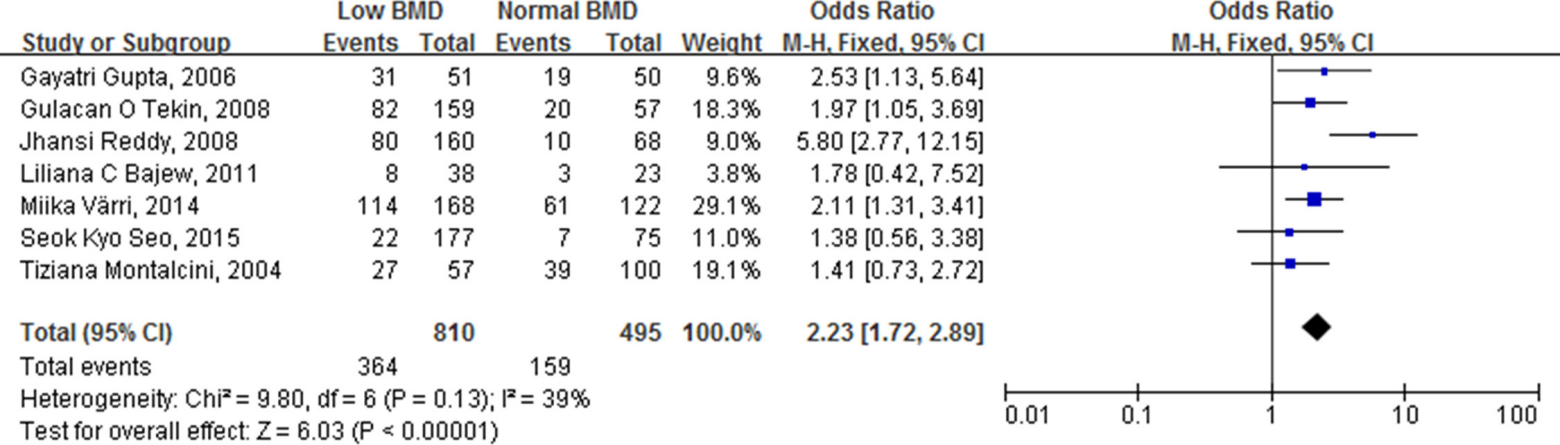
Forest plot shows that the incidence of atherosclerotic vascular abnormalities is significantly higher in postmenopausal women with low BMD than those with normal BMD.

**Fig 5:**
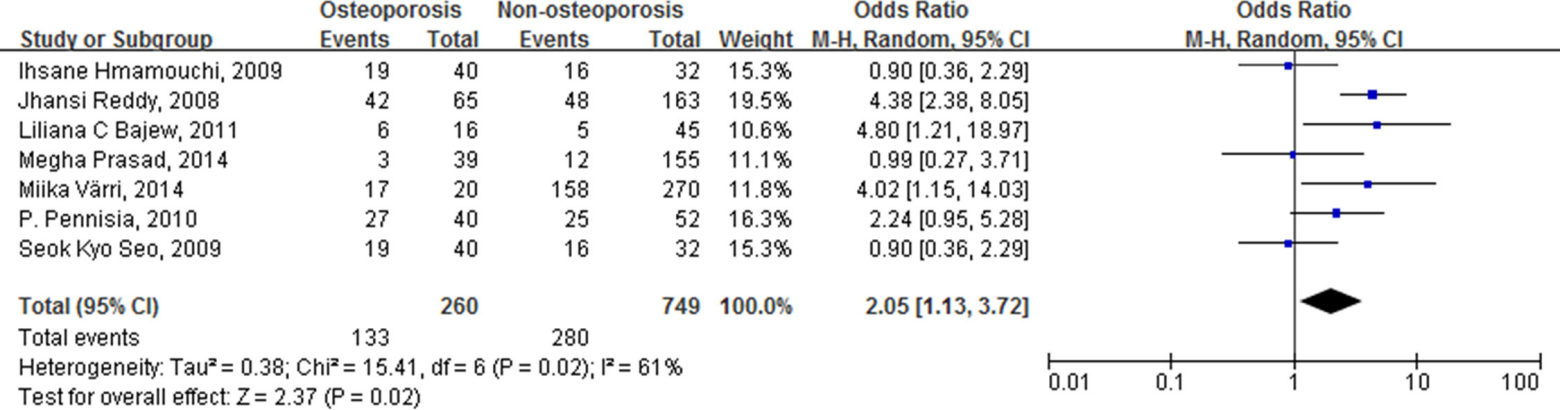
Forest plot shows that the incidence of atherosclerotic vascular abnormalities is significantly higher in postmenopausal women with osteoporosis than those without osteoporosis.

When including only postmenopausal women, the combined ORs for the incidence of atherosclerotic vascular abnormalities in patients with low BMDs, versus patients with normal BMDs, was 2.23 (95% CI [1.72, 2.89], p < 0.00001) (Fig 4). When comparing patients with osteopenia to those with normal BMDs, the combined OR was 2.07 (95% CI [1.43, 3.00], p < 0.00001). For patients with osteoporosis versus patients with normal BMD, the combined OR was 4.29 (95% CI [2.37, 7.77], p < 0.00001). Comparing patients with osteoporosis to patients without, the combined OR for the incidence of atherosclerotic vascular abnormalities was 2.05 (95% CI [1.13, 3.72], p = 0.02) (Fig 5).

Thus, the results revealed that under all circumstances, the combined OR for the incidence of atherosclerotic vascular abnormalities increased as BMD decreased.

### Adjusted results

**Fig 6:**
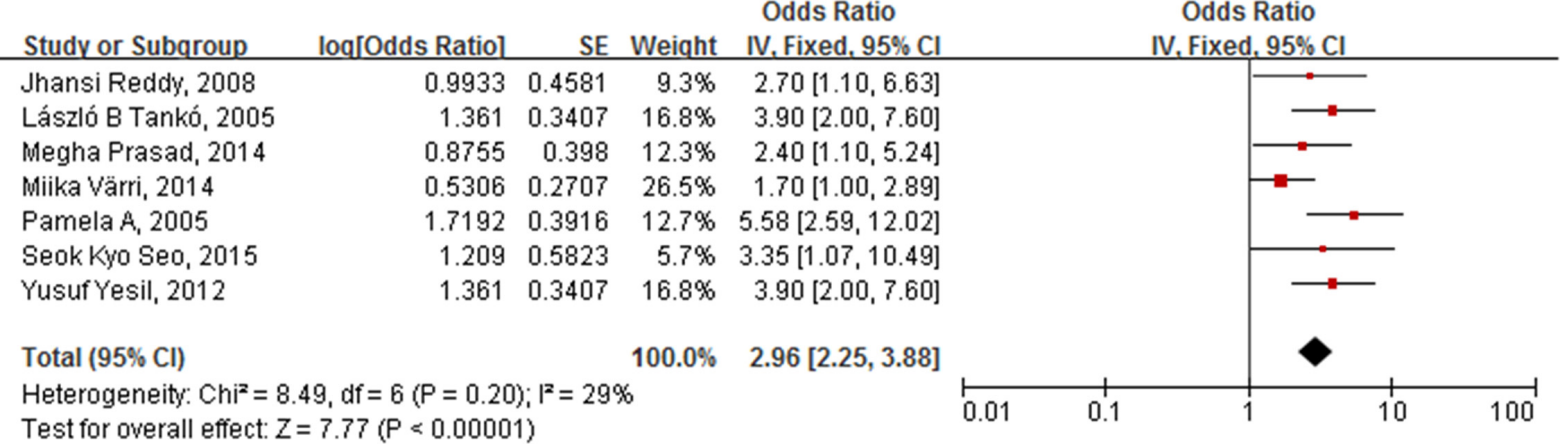
Forest plot shows that the incidence of atherosclerotic vascular abnormalities is significantly higher in individuals with low BMD than those with normal BMD, after adjusting for age, gender, BMI, hypertension, and other vascular risk factors.

After adjusting for age, gender, BMI, hypertension, and other vascular risk factors, the combined OR for the incidence of atherosclerotic vascular abnormalities in patients with low BMD versus patients with normal BMD was 2.96 (95% CI [2.25, 3.88], p < 0.00001; I^2^ = 29%) (Fig 6).

### Publication bias

**Fig 7:**
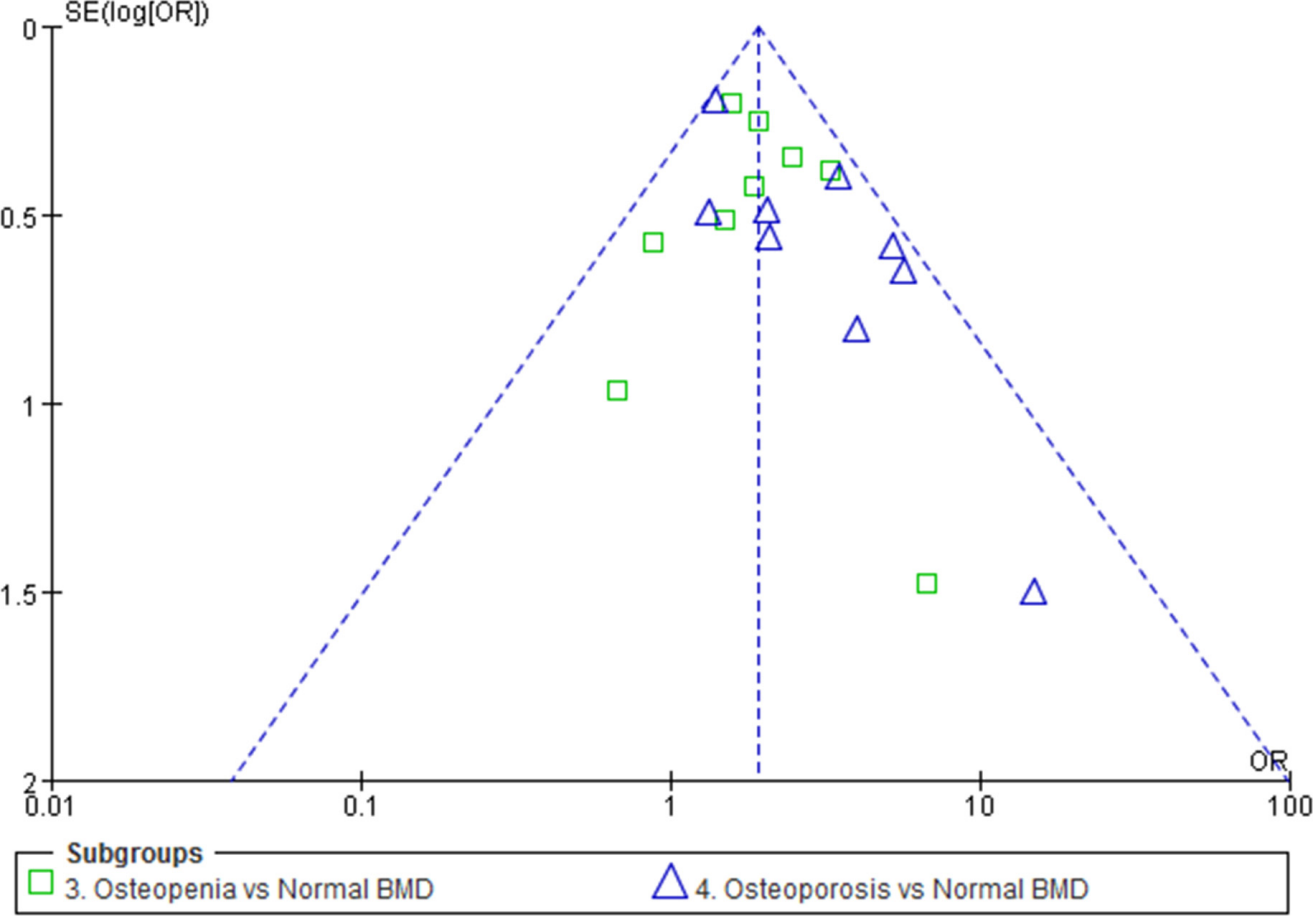
Funnel plot of combined OR for patients with low BMD compared with patients normal BMD showed no publication bias in visual.

No substantial asymmetry was identified using Begg's rank correlation test (z = 1.25, p = 0.211) and Egger's regression test (t = 1.38, p = 0.187). Funnel plots of the combined ORs for patients with low BMDs, compared to patients with normal BMDs also showed no publication bias (Fig 7).

## Discussion

The systematic review and meta-analysis presented here revealed a strong link between decreased bone mineral density and the risk of atherosclerotic vascular abnormalities. This finding was independent of age, gender, BMI, hypertension, and other vascular risk factors. The combined ORs increased as BMD decreased. Moreover, the link between deceased bone mineral density and atherosclerotic vascular abnormalities was not only seen in postmenopausal women, but also in elderly men (aged above 50 years). These findings suggest that postmenopausal women and elderly men with decreased BMD have a higher risk of developing atherosclerotic vascular abnormalities. Our results not only confirmed a significant relationship between osteoporosis and atherosclerotic vascular abnormalities, as previous studies have suggested [3, 4, 6–13], but also a relationship between osteopenia and atherosclerotic vascular abnormalities. Based on the results of our study, patients who have decreased BMD scores, should be screened for cardiovascular risk, as it is an independent indicator of significant atherosclerotic burden. Besides, an enhanced understanding of the relationship between decreased BMD and atherosclerosis might lead to the development of therapeutic agents for the prevention and treatment of both osteoporosis and atherosclerotic vascular abnormalities.

In this study, no evidence of significant heterogeneity was observed between studies, except for the comparison between postmenopausal women with and without osteoporosis. The following reasons may account for this finding: the definition of atherosclerotic vascular abnormalities is clear and the severity of bone loss is definite, according to the standard of the WHO. Moreover, we included only high quality studies with a NOS score of five or more (S5 Text). In the subgroup analysis of postmenopausal women with and without osteoporosis, medium heterogeneity (I^2^ = 61%) was observed. When excluding the data of Reddy *et al*. [29], which used the breast arterial calcification (BAC) as a defined index of atherosclerotic vascular abnormalities, the heterogeneity reduced to 41% (p = 0.13).

The key pathology in atherosclerotic vascular abnormalities is the formation of calcified plaque and chronic inflammation on the endothelial surface, which progressively leads to plaque rupture and thrombosis (S3 Text)[52–55]. Superficial erosion and fibrous cap rupture are the two possible mechanisms of plaque disruption [54–56]. Inflammatory processes of the vascular wall should play a particularly important role in regulating the integrity of the fibrous cap. It is reported that inflammatory cytokines produced by leukocytes increase degradation of the structural components of the fibrous cap, which further increase the vulnerability of the plaque to rupture [57, 58]. Moreover, biomechanical studies showed that the increased circumferential stress also decrease the stability of the atherosclerotic lesion and increase the incidence of plaque rupture and thrombosis (S3 Text)[59].

Based on multiple biological observations, arterial vascular calcification is highly regulated and organized by mechanisms similar to those involved in bone mineralization [60, 61]. Additionally, several bone matrix proteins, including matrix Gla protein (MGP), osteoprotegerin (OPG), osteopontin (OPN), osteonectin (ON), bone morphogenetic protein (BMP)-2, collagen I, osteocalcin (OC), receptor-activated nuclear factor-kappa B ligand (RANKL), and the mineral hydroxyapatite, also exist in atherosclerotic arteries [62–66]. Inflammatory cytokines like interleukin-1 (IL-1), interleukin-1 (IL-6), tumor necrosis factor (TNF), and osteoclast-like cells (OLCs) have also been reported in calcified arteries (S3 Text)[67].

OPG/RANK/RANKL system is reported to play a key role in calcium deposition and bone formation [68]. RANKL is mainly expressed by osteoblast precursors. The combination between RANKL and RANK promote osteoclast formation and lead to bone resorption [69]. OPG is mainly produced by osteoblasts, and vascular smooth muscle cells (VSMCs), and is a potent inhibitor of osteoclast activation [70]. It is reported that in patients with atherosclerotic vascular abnormalities, OPG is over expressed lining the calcific deposits, while RANKL is expressed in the extracellular matrix around the calcific lesions. The OPG-knockout mice developed both osteoporosis and arterial calcification [71]. These findings suggest that the possible mechanism of OPG/RANK/RANKL on atherosclerosis may due to its differential and site-specific expression [72].

MGP and OC are both Gla-containing proteins that inhibit osteoid formation. It is reported that MGP and OC are both expressed in bone and vascular wall and are both up-regulated in atherosclerotic vessels. Furthermore, as key inhibitors of arterial calcification, MGP and OC inhibit BMP-induced chondrocyte differentiation and osteogenic differentiation of the vascular mesenchyme [73]. In animal experiments, knockout mice models of MGP, displayed both vascular calcification and osteoporosis [60]. In women with osteoporosis and atherosclerosis, serum MGP and OC are also elevated [74]. The possible mechanisms of other bone matrix proteins were shown in (S4 Text), including OPN, ON, BMP etc., which help providing an overview regarding the pathogenesis of concomitant osteoporosis and atherosclerosis.

Besides the potential mechanism introduced above, some characteristics may also influence the risk of atherosclerotic vascular abnormalities in postmenopausal women. Estrogen can prevent the development of atherosclerotic vascular abnormalities. For postmenopausal women, deficiency of estrogen not only impairs normal bone remodeling by breaking the balance of osteoblasts, but also increases the risk of cardiovascular diseases (CAD) [75]. Free iron deposits in tissues, which are responsible for iron overload, also play an important role in inhibiting osteoblast proliferation, which impairs bone remodeling [75]. Postmenopausal women showed a stronger correlation between low BMD and the development of atherosclerotic vascular abnormalities [3, 11, 76, 77]. In a study by Sung *et al*. [78], the authors showed that both premenopausal and postmenopausal women had a correlation between low BMD and atherosclerotic vascular abnormalities.

Uyl et al. conducted a systematic review in 2011 and indicated that persons with prevalent subclinical CV disease are at increased risk for bone loss, however, the association between different extent of low bone mineral density and increased cardiovascular risk was still not clear [7]. To clarify it, we included 25 studies with a NOS score ≥ 5 points and compared the incidence of atherosclerotic vascular abnormalities in people with different extent of decreased BMD. To our knowledge, this is the first systematic review and meta-analysis comparing the incidence of atherosclerotic vascular abnormalities in individuals with normal BMD, osteopenia and osteoporosis. Moreover, it is a relatively comprehensive and up-to-date summary of data on the topic. Male and female participants were included, and gender-specific analyses were performed in the subgroup analysis. Compared to the original studies, the large number of cases and participants increased the detection of significant associations and provided more precise estimates of their effects.

This systematic review and meta-analysis also has several limitations. Because of the paucity of randomized controlled trials, this study only evaluated case-control, cross-sectional and cohort studies, which decreases the robustness of the conclusions. Secondly, most of the included studies were based on healthy adults, which can limit the generalizability of the findings to patients with multiple atherosclerosis risk factors and comorbidities. Although we managed to conduct subgroup analyses regarding different severity of decreased BMD, we had limited ability to assess the relationship between different pathologic stages of atherosclerotic vascular abnormalities and decreased bone mineral density due to the lack of available studies. Future quantitative research focusing on individuals with different severities of atherosclerotic vascular abnormalities, and patients with different comorbidities, will be of interest.

## Conclusion

Based on the results of this study, decreased BMD is an independent predictor for the development of atherosclerosis in elderly individuals. Additionally, the risk increases as BMD decreases. Patients who have decreased BMD scores, should be screened for cardiovascular risks. Future studies focusing on individuals with different severities of atherosclerotic vascular abnormalities and patients with different comorbidities would be of interest.

## Supporting Information

S1 PRISMA Checklist PRISMA 2009 Checklist. (DOC)

S1 Table Search strategy for PubMed. (DOCX)

S1 Text Search strategy for Embase. (DOCX)

S2 Text Search strategy for Cochrane Library. (DOC)

S3 Text Mechanisms of the development of atherosclerosis and plaque rupture. (DOC)

S4 Text Common cellular mechanisms linking atherosclerotic vascular disease and osteoporosis. (DOC)

S5 Text Subgroup analysis based on the quality of included studies. (DOC)
