# Supplementary material for: Decreased Bone Mineral Density Is an Independent Predictor for the Development of Atherosclerosis: A Systematic Review and Meta-Analysis
Source: PLoS One. 2016 May 5;11(5):e0154740. doi: 10.1371/journal.pone.0154740 (PMC4858264; doi:10.1371/journal.pone.0154740)

| **Search strategies in Pubmed: #3** |
| --- |
| #3  Search ((((((bone mineral density[Title/Abstract]) OR BMD[Title/Abstract]) OR osteoporosis[Title/Abstract]) OR OP[Title/Abstract]) OR osteopenia[Title/Abstract])) AND (((((((((((((atherosclerosis[Title/Abstract]) OR atheroscleroses[Title/Abstract]) OR calcium plaque[Title/Abstract]) OR carotid artery calcification[Title/Abstract]) OR CAC[Title/Abstract]) OR cardiovascular disease[Title/Abstract]) OR CVD[Title/Abstract]) OR coronary artery disease[Title/Abstract]) OR CAD[Title/Abstract]) OR atherosclerotic vascular disease[Title/Abstract]) OR AVD[Title/Abstract]) OR coronary micro-vascular endothelial dysfunction[Title/Abstract]) OR CMED[Title/Abstract])  #2  Search ((((((((((((atherosclerosis[Title/Abstract]) OR atheroscleroses[Title/Abstract]) OR calcium plaque[Title/Abstract]) OR carotid artery calcification[Title/Abstract]) OR CAC[Title/Abstract]) OR cardiovascular disease[Title/Abstract]) OR CVD[Title/Abstract]) OR coronary artery disease[Title/Abstract]) OR CAD[Title/Abstract]) OR atherosclerotic vascular disease[Title/Abstract]) OR AVD[Title/Abstract]) OR coronary micro-vascular endothelial dysfunction[Title/Abstract]) OR CMED[Title/Abstract]  #1  Search ((((bone mineral density[Title/Abstract]) OR BMD[Title/Abstract]) OR osteoporosis[Title/Abstract]) OR OP[Title/Abstract]) OR osteopenia[Title/Abstract] |
|  |
|  |


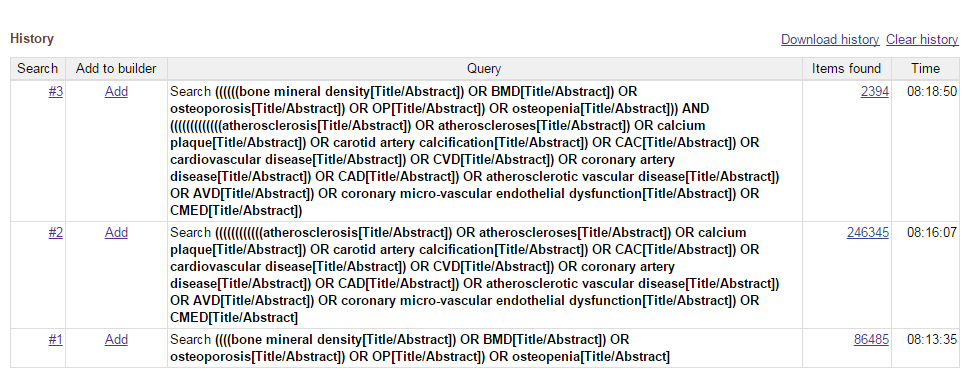

Supplement: S1 Table — (DOCX) [file pone.0154740.s002.docx]
