# Supplementary material for: Decreased Bone Mineral Density Is an Independent Predictor for the Development of Atherosclerosis: A Systematic Review and Meta-Analysis
Source: PLoS One. 2016 May 5;11(5):e0154740. doi: 10.1371/journal.pone.0154740 (PMC4858264; doi:10.1371/journal.pone.0154740)

**Embase search strategy**

**#3** **#1** combined **#2**

**#2** 'bone mineral density' OR 'bmd' OR 'osteoporosis' OR 'op' OR 'osteopenia'

**#1** 'calcium plaque' OR 'atherosclerosis'/exp OR 'atherosclerosis' OR 'carotid artery calcification' OR 'cardiovascular disease'/exp OR 'cardiovascular disease' OR 'coronary artery disease'/exp OR 'coronary artery disease' OR 'atherosclerotic vascular disease'


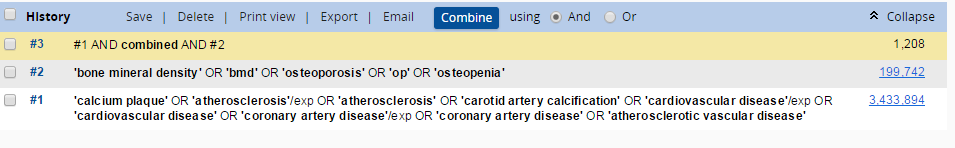

Supplement: S1 Text — (DOCX) [file pone.0154740.s003.docx]
