# Supplementary material for: Decreased Bone Mineral Density Is an Independent Predictor for the Development of Atherosclerosis: A Systematic Review and Meta-Analysis
Source: PLoS One. 2016 May 5;11(5):e0154740. doi: 10.1371/journal.pone.0154740 (PMC4858264; doi:10.1371/journal.pone.0154740)

**Search strategies in the Cochrane Library:**

#1

"bone mineral density":ti,ab,kw or "BMD":ti,ab,kw or "osteoporosis":ti,ab,kw or "OP":ti,ab,kw or osteopenia:ti,ab,kw (Word variations have been searched)

#2

"atherosclerosis":ti,ab,kw or "atheroscleroses":ti,ab,kw or coronary artery disease:ti,ab,kw or "atherosclerotic vascular disease":ti,ab,kw or cardiovascular disease:ti,ab,kw (Word variations have been searched)

#3

#1 and #2


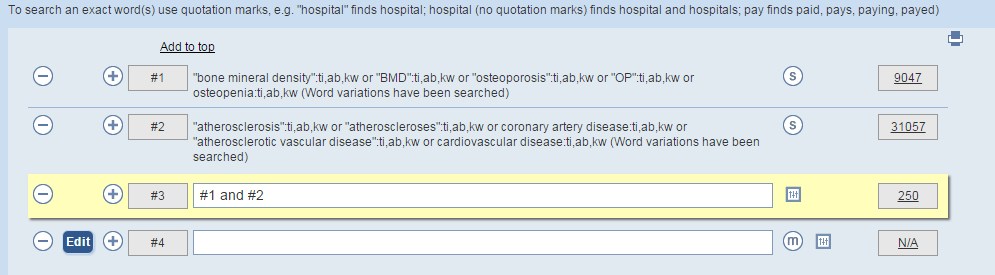

Supplement: S2 Text — (DOC) [file pone.0154740.s004.doc]
