# Supplementary figures and images for: Decreased Bone Mineral Density Is an Independent Predictor for the Development of Atherosclerosis: A Systematic Review and Meta-Analysis
Source: PLoS One. 2016 May 5;11(5):e0154740. doi: 10.1371/journal.pone.0154740 (PMC4858264; doi:10.1371/journal.pone.0154740)

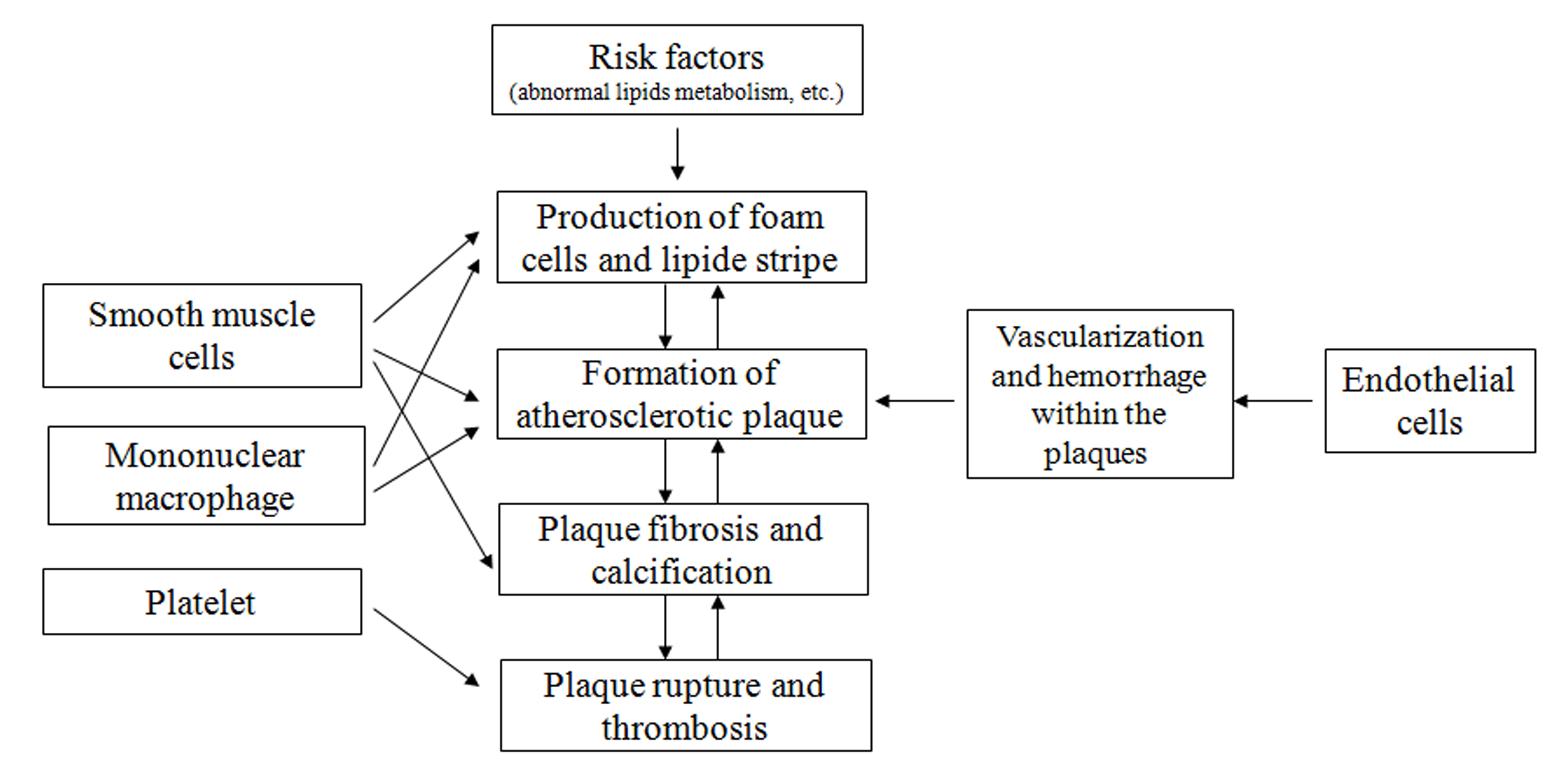


S3. Mechanisms of the development of atherosclerosis and plaque rupture.

Supplement: S3 Text — (DOC) [file pone.0154740.s005.doc]
