# Supplementary material for: Decreased Bone Mineral Density Is an Independent Predictor for the Development of Atherosclerosis: A Systematic Review and Meta-Analysis
Source: PLoS One. 2016 May 5;11(5):e0154740. doi: 10.1371/journal.pone.0154740 (PMC4858264; doi:10.1371/journal.pone.0154740)
Supplement: S4 Text — (DOC) [file pone.0154740.s006.doc]

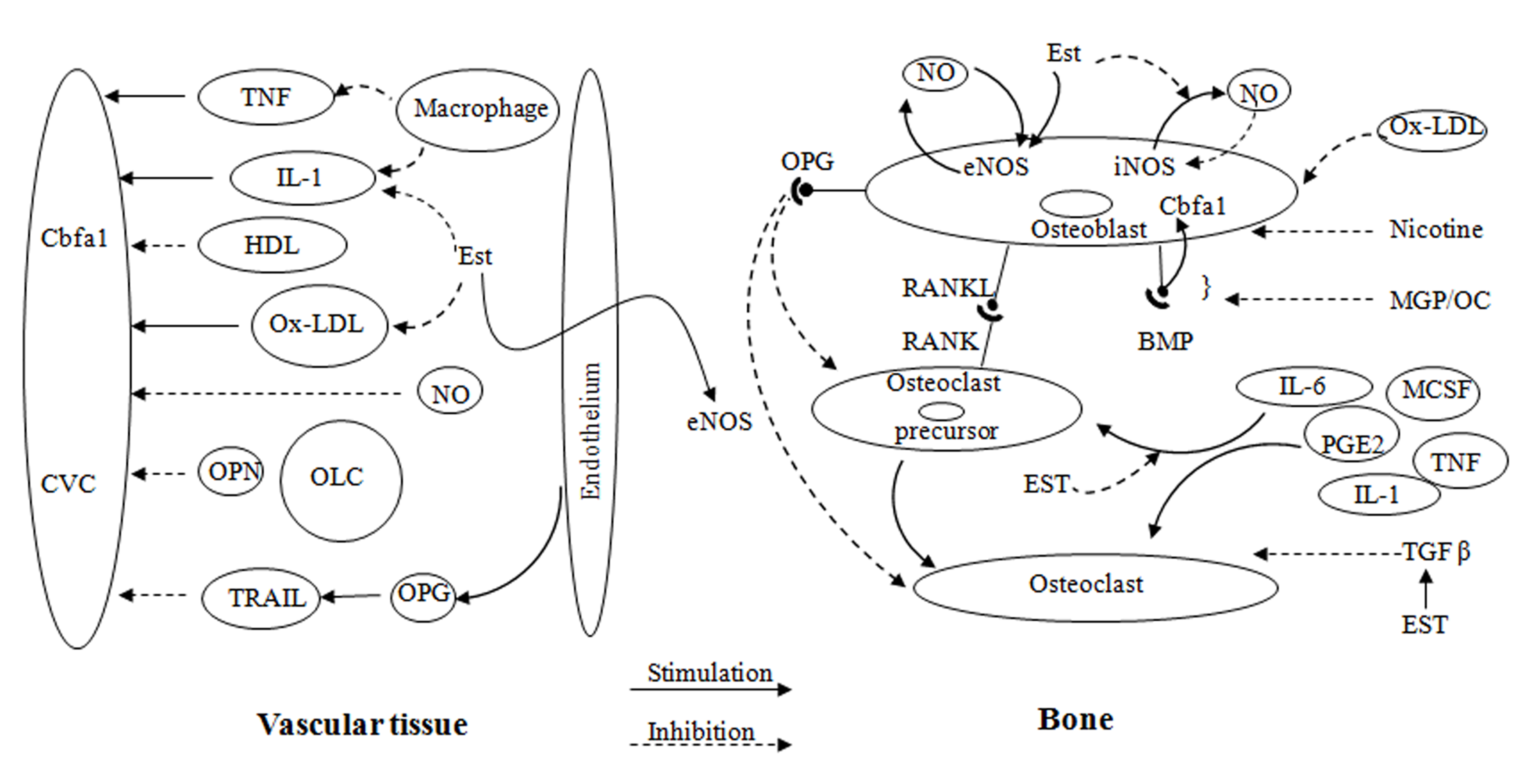


S4. Common cellular mechanisms linking atherosclerotic vascular disease and osteoporosis. eNOS = endothelial nitric oxide synthase; Est = estrogen; NO = nitric oxide; INOS = inducible nitric oxide synthase; HDL = high-density lipoprotein; Ox-LDL = oxidized-low-density lipoprotein; RANK = receptor-activated nuclear factor-kappa B; RANKL = receptor-activated nuclear factor-kappa B ligand; OPG = osteoprotegerin; MGP = matrix Gla protein; M-CSF = macrophage-colony-stimulating factor; BMP = bone morphogenetic protein; OC = osteocalcin; IL-1 = interleukin-1; IL-6 = interleukin-6; TNF = tumor necrosis factor; TGFβ = transforming growth factor-beta; PGE2 = prostaglandin E2; OPN = osteopontin; OLC = osteoclast-like cell; CVC = calcifying vascular cell; TRAIL = tumor necrosis factor-related ligand; Cbfα-1 = core-binding factor-1.
