# Supplementary material for: Decreased Bone Mineral Density Is an Independent Predictor for the Development of Atherosclerosis: A Systematic Review and Meta-Analysis
Source: PLoS One. 2016 May 5;11(5):e0154740. doi: 10.1371/journal.pone.0154740 (PMC4858264; doi:10.1371/journal.pone.0154740)
Supplement: S5 Text — (DOC) [file pone.0154740.s007.doc]

S5. Subgroup analysis based on the quality of included studies.


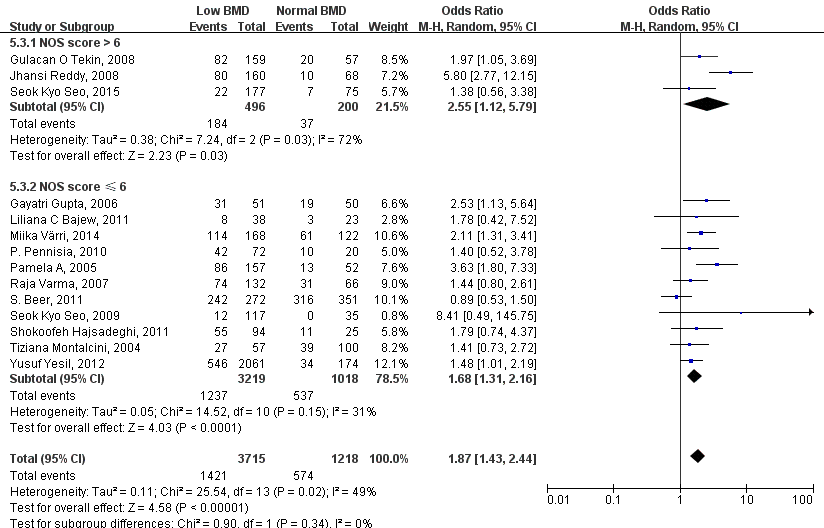


Fig. 1. Forest plot shows that the incidence of atherosclerotic vascular abnormalities is significantly higher in individuals (including male and female) with low BMD than those with normal BMD.


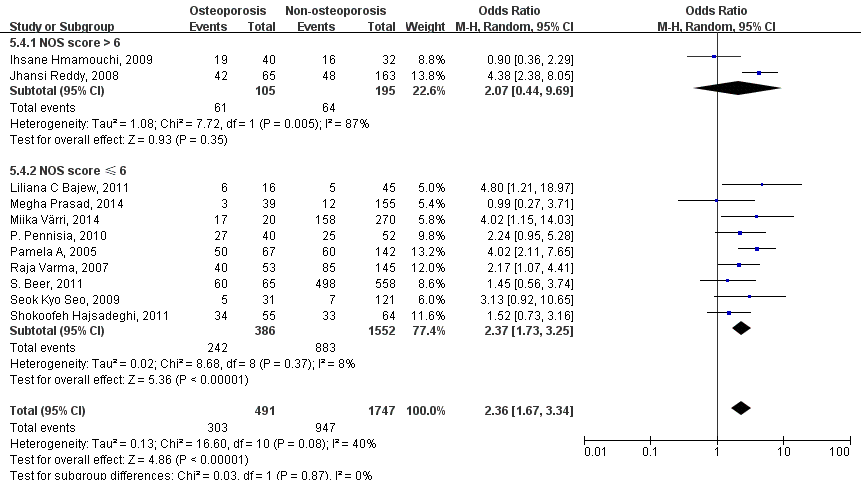


Fig. 2. Forest plot shows that the incidence of atherosclerotic vascular abnormalities is higher in individuals (including male and female) with osteoporosis than those without osteoporosis.


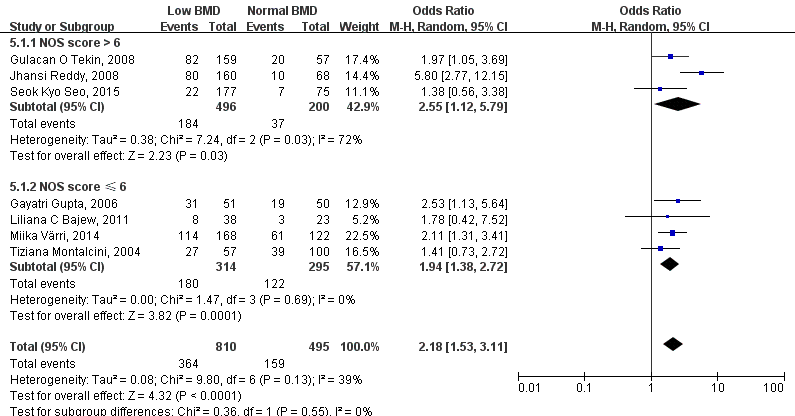


Fig. 3. Forest plot shows that the incidence of atherosclerotic vascular abnormalities is significantly higher in postmenopausal women with low BMD than those with normal BMD.


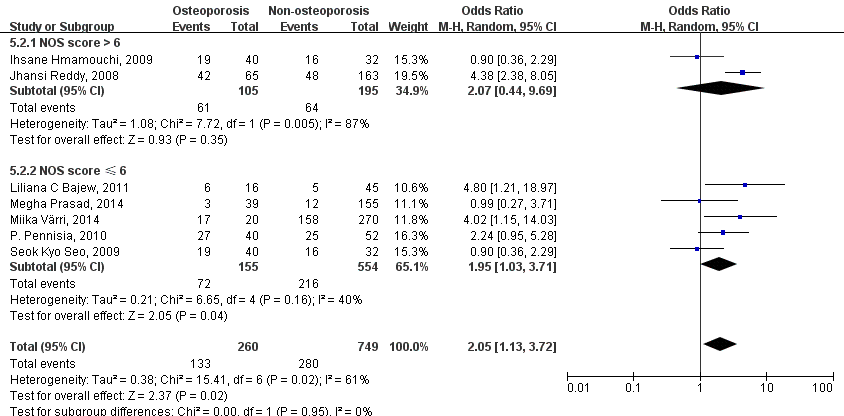


Fig. 4. Forest plot shows that the incidence of atherosclerotic vascular abnormalities is higher in postmenopausal women with osteoporosis than those without osteoporosis.


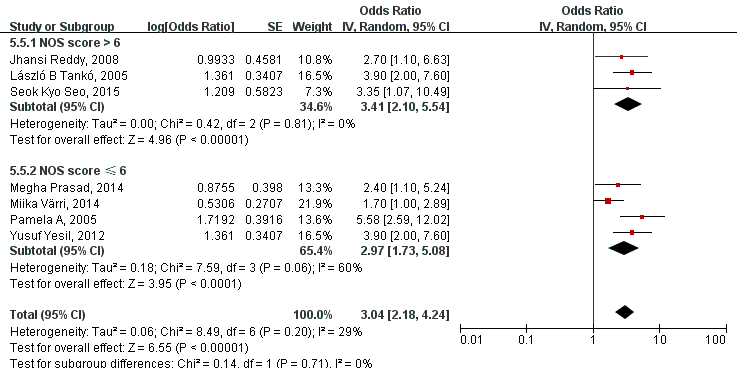


Fig. 5. Forest plot shows that the incidence of atherosclerotic vascular abnormalities is significantly higher in individuals with low BMD than those with normal BMD, after adjusting for age, gender, BMI, hypertension, and other vascular risk factors.
